# The Use of Critical Levels for Determining Plant Response to Ozone in Europe and in North America

**DOI:** 10.1100/tsw.2007.24

**Published:** 2007-03-21

**Authors:** Robert C. Musselman, Allen S. Lefohn

**Affiliations:** ^1^ USDA Forest Service, Rocky Mountain Research Station, Fort Collins, CO, USA; ^2^ A.S.L. and Associates, Helena, MT, USA

**Keywords:** AOT40, critical levels, detoxification, effective flux, flux, National Ambient Air Quality Standards, ozone, SUM06, W126

## Abstract

Critical levels to determine plant response to ozone (O_3_) have been used in Europe since the 1980s, utilizing the concentration-based AOT40 to relate plant response to ambient O_3_ exposure. More recently, there has been progress in Europe toward utilizing flux-based critical levels, because plant response is more closely related to O_3_ uptake than to the amount of O_3_ in ambient air. Flux-based critical levels are plant species specific; data for parameterization of flux-based critical levels models are lacking for most plant species. Although flux-based critical levels are now being used for a limited number of agricultural crops and tree species where data are available, the use of flux-based critical levels is limited by the lack of adequate consideration and incorporation of plant internal detoxification mechanisms in flux modeling. Critical levels have not been used in North America; however, recent interest in the U.S. and Canada for using critical loads for nitrogen and sulfur has generated interest in using critical levels for O_3_. A major obstacle for utilization of critical levels in North America is that ambient air quality standards for O_3_ in the U.S. and Canada are concentration based. It appears that cumulative exposure-based metrics, particularly when implemented with a quantification of peak concentrations and environmental variables, such as a drought index, are currently the most useful to relate O_3_ to vegetation response. Because data are unavailable to quantify detoxification potential of vegetation, effective flux models are not available to determine plant response to O_3_.

Ambient levels of ozone ($O_3$) have been shown to impact vegetation in North America and in Europe, causing leaf necrosis and reductions in growth and yield of agricultural crops, forest trees, and natural vegetation. It is important to be able to quantify the relationship between ambient $O_3$ exposure and plant response for policy makers to determine effectiveness of emission controls designed to benefit vegetation. Assessing this impact has been difficult. Traditionally, $O_3$-caused necrosis on plant tissue, primarily oxidant stipple necrosis, has been used as an indicator of negative $O_3$ impact on plant tissue. Attempts to relate the amount of visible injury occurring to various $O_3$ summary metrics has not met with much success.

The relationship between $O_3$-induced necrotic injury (leaf necrosis) on plant tissue and plant damage (loss of yield, see Musselman et al.[1] for definitions of injury and damage) has been weak. Plants can show considerable amounts of $O_3$-induced leaf necrosis, such as oxidant stipple, with little effect on plant growth or yield. Conversely, plant growth and/or yield can be reduced with little or no oxidant stipple or other $O_3$-induced leaf necrosis or chlorosis. Plant response to $O_3$ is highly dependent on plant genetics, with some plant species or varieties within species susceptible and some tolerant to $O_3$-induced leaf necrosis or growth and yield reduction. Complicating the matter further, environmental conditions when the plants are exposed, particularly the amount of plant water stress, will influence plant response to $O_3$. Reduction in stomatal uptake due to stomatal closure occurs during periods of water stress. This additional stress also may interfere with the ability of plant to cope with an additional oxidative stress such as $O_3$.

Research has shown that ambient $O_3$ summary metrics that most closely relate to plant response are those that preferentially weight the higher concentrations, are cumulative over a growing season, and include the time period during the day when stomata are open and $O_3$ uptake into plant tissue is possible[1]. Concentration is the number of moles per unit volume of air. Common $O_3$ concentration metrics that are used to relate $O_3$ to plant response are the maximum daily hourly $O_3$ concentration (P1) during a specific period of time, the number of $O_3$ hourly concentrations $\geq$ 100 ppb (N100) during a specific period of time, and the fourth highest daily maximum concentration averaged over a 3-year period (4thHDM). Exposure is some product of the concentration to which the vegetation of interest is exposed and the length of time that vegetation is presumably exposed to the pollutant[1]. However, in order to avoid the inappropriate use of Haber's Law (i.e., where the product of concentration multiplied by time predicts a constant effect), the exposure usually includes a threshold- or weighting-based scheme to reduce the importance of the lower hourly average concentrations.

The $O_3$ cumulative exposure summary parameters most frequently used to relate to plant response have been the SUM06 and W126 in North America and the AOT40 in Europe. The SUM06 is the sum of the hourly average concentrations $\geq$ 0.06 ppm, the W126 is a sigmoidally weighted function that preferentially weights the higher concentrations up to 0.1 ppm, and the AOT40 accumulates all concentrations after subtracting 40 ppb from each hourly value. All three exposure metrics are an accumulation of $O_3$ concentration values over a specific period of time, often a growing season. The SUM06 and AOT40 summary statistics somewhat weight the higher concentrations by being threshold metrics; the SUM06 by eliminating all hourly concentrations less than 60 ppb, the AOT40 includes only those hourly concentrations greater than 40 ppb. The W126 weights the higher concentrations by increasingly weighting all concentrations as they increase up to 0.1 ppm, but does not discard the lower concentrations as does the SUM06 and AOT40. The W126 ($W = 1/[1+4403 \cdot e^{-(126 \cdot Ci)}]$ weights the lower concentrations less, but does not ignore them. Although the 40-ppb level of the AOT40 was selected as a cut-off to differentiate from assumed background $O_3$ concentrations, the elimination of the lower concentrations effectively assumes that there is a threshold for plant response and the lower $O_3$ concentrations have no negative effects on plant tissue. Similarly, the elimination of all values below 60 ppb for the SUM06 effectively eliminates the effect of all $O_3$ concentrations less than 60 ppb. The threshold theory assumes that detoxification eliminates these initial $O_3$ molecules and it takes a loading to the 40- or 60-ppb level before detoxification becomes overwhelmed and a negative impact on the plant is realized. However, Musselman et al.[1] point out that detoxification is a time-varying process and the selection of a specific threshold is not necessarily appropriate. It is expected that any $O_3$ molecule contacting leaf tissue requires a plant response that expends plant energy[1,2].

The SUM06 and W126 as used in the U.S. sometimes utilize data from 0800–1959 h to capture the time period during the day when stomata are most open, while the AOT40 uses actual daylight hours. However, uptake at night should not be ignored[1,3]. Nighttime stomatal uptake, though less than daytime uptake, can be a significant amount of $O_3$[4,5]. The reduced uptake at night is not necessarily linearly related to less impact, because antioxidants cannot be replenished without photosynthetic activity. Detoxification of $O_3$ may be less at night compared to daytime. The result is that significant impact might occur during the nighttime hours.

The $O_3$ concentration values used in North American vegetation assessments are hourly averages, since almost all ambient $O_3$ data in North America are summarized in $O_3$ databases as hourly averages. These data are derived from continuous monitoring, but data are almost always logged and summarized as hourly averages. Plants can be exposed to short-time $O_3$ concentrations considerably higher than hourly averages, and these higher exposures may be important in plant response; nevertheless, because almost all $O_3$ data are reported as hourly averages, these data are necessarily used in calculation of $O_3$ concentration and exposure metrics. The usefulness of passive $O_3$ data, one-time accumulated loading data over a 1- or 2-week exposure, has been limited to those sites where the passive data are calibrated to active, continuous monitoring data. Because passive monitors integrate over a long time period, they provide "averaged" information that cannot be used to characterize the pattern of the higher hourly average concentrations.

Cumulative exposure metrics, such as the SUM06, W126, and AOT40, when used alone, are inadequate to characterize $O_3$ exposure of vegetation. For example, similar SUM06 (or SUM07) values with differing peak concentrations have shown different injury[6] or yield[7] response. Musselman et al.[1] have shown that two areas with similar SUM06 values can experience considerably different $O_3$ exposures. This is because the identical SUM06 value can consist of a large number of peak $O_3$ concentrations and moderate number of mid-level values, or an infrequent number of peak values and a large number of mid-level $O_3$ concentrations. The inclusion of the N100 has been recommended in combination with the SUM06 or W126 exposure metrics to quantify the peak concentration exposures and improve on the predictive ability of the cumulative exposure indices[1,8,9]. The inclusion of the N100 would quantify those peak concentrations that have been reported to be so important to plant response[10].

The exposure parameters SUM06, W126, or AOT40 used alone or with the N100 may not necessarily be adequate for determining plant response to $O_3$. The amount of $O_3$ taken up into plant tissue may not be closely related to the amount to which the plant is exposed and available in the ambient air for uptake. Ozone uptake is species dependent and is determined by stomatal conductance. It might be expected that flux dose metrics that consider $O_3$ uptake might be more related to plant response than exposure metrics. However, once $O_3$ is taken up into the leaf, the internal cellular response and detoxification determines the amount of $O_3$ interaction with plant tissue for injury and damage. Although it seems that $O_3$ taken up into the leaf should be more related to plant response than ambient exposure to $O_3$, without detoxification potential included with the uptake parameter, uptake will not adequately predict plant response.

## CRITICAL LOADS/LEVELS IN EUROPE

European evaluation of $O_3$ impacts to vegetation is different than the standard-setting process in North America. In contrast to the U.S. process of the determination of critical concentrations of ambient $O_3$ that might be harmful, the European process for planning reductions in emissions attempts to identify the lowest critical level of $O_3$ exposure or dose that has a negative effect on the most sensitive vegetation receptor. Critical levels have been used in Europe since the 1980s to assess $O_3$ effects on crops, forests, and natural vegetation. A critical level is the "concentration, cumulative exposure or cumulative stomatal flux of atmospheric pollutants above which direct adverse effects on sensitive vegetation may occur according to present knowledge"[11]. Concentration-based (previously called Level I) critical levels are based on exposure of vegetation to ambient $O_3$ (i.e., concentration over a period of time). The $O_3$ summary statistic for the concentration-based assessment is the AOT40. Although the AOT40 is called a concentration-based parameter, it is accumulated over a 3- (crops) or 6-month (forest trees) period of time; thus, it is an exposure-based parameter. Concentration-based critical levels have been determined for crops, forest trees, and natural vegetation, generally determined from semi-controlled environmental studies looking at growth and yield response of specific crops or trees to specific $O_3$ concentration exposure levels. However, it has been noted that they provide "an incorrect assessment of the regional distribution of the risk of damage to vegetation by $O_3$ across Europe"[12]. Combining the AOT40 with soil water availability has been recommended to better estimate effects of ozone on wheat yield in Europe[13].

Flux-based (previously called Level II) critical level assessments “offer the potential for improved quantitative evaluation of the impacts of $O_3$ on vegetation across Europe”[12] and are based on the Jarvis[14] model using a multiplicative algorithm of stomatal conductance of $O_3$[15]. These models include plant species–specific data on conductance and phenology, and include environmental data on light, temperature, vapor pressure deficit, and soil water potential. However, detoxification potential is not included in the models at this time. Data are available to determine flux-based assessments for two crops in Europe, wheat and potato[16]. Provisional flux-based assessments are also available for beech and birch trees[17]. Additional flux-based models have been parameterized for grapevine, sunflower, tomato, and maize, but datasets to determine flux-based relationships are unavailable[18].

## CRITICAL LOADS/LEVELS IN THE U.S. AND CANADA

Efforts have recently been made to determine critical loads for nitrogen and sulfur for ecosystems in the U.S.[19,20]. These efforts have not used the European critical loads Simple Mass Balance (SMB) or Very Simple Dynamic (VSD) models[12]. Others have begun to use the European models to determine critical loads in the U.S. and Canada[21]. Data for input to these analyses have been gathered where available, sometimes from USDA Forest Service Forest Health Monitoring (FHM) plots in the U.S., but specific locations of individual plots are unavailable. The U.S. has yet to establish on-the-ground ICP-protocol plots designed specifically to generate data for input to the critical loads models. Thus, critical loads and critical levels analyses, using the models developed and tested in Europe, are rare in North America. Occasionally, concentration-based AOT40 data have been determined for specific areas along with W126 or SUM06 data, and the AOT40 metric has been compared with the W126 and SUM06 metrics in examination of plant response. Flux-based critical level assessments have not been conducted in North America. Plant-specific and environmental data necessary for input into flux models are essentially nonexistent in North America.

The U.S. Clean Air Act (CAA) establishes National Ambient Air Quality Standards (NAAQS) for $O_3$. In the U.S., both a primary standard (public health) and secondary standard (public welfare, including soils, water, crops, and vegetation) are established. Since the first $O_3$ NAAQS was established in 1971, the primary and secondary standards have been the same because it was believed that the primary standard was stringent enough to protect both human health and vegetation. The current NAAQS is supposed to be re-evaluated every 5 years. After the first $O_3$ NAAQS were established, re-evaluations were completed in 1979, 1993, and 1997[10]. A current $O_3$ re-evaluation is underway and is expected to be completed in 2007. The re-evaluation process prior to December 2006 involved four steps. First, an Air Quality Criteria Document (AQCD) was prepared. This was an extensive peer-reviewed synthesis of the literature detailing the latest state of the science on the issue. Second, the U.S. EPA Office of Air Quality Planning and Standards (OAPQS) staff prepared the Staff Report, an assessment of the current standard and any recommended changes, based on the AQCD and other information staff might obtain to assess the adequacy and need to modify the current standard. Third, the Clean Air Science Advisory Committee (CASAC) independently evaluated the adequacy of the AQCD and then the Staff Report, and provided its recommendation to the EPA Administrator. The 1996 and 2006 AQCDs, 2006 Staff Paper, and current CASAC recommendations are available online (http://www.epa.gov/ttn/naaqs). Finally, the EPA Administrator issues a decision on the latest NAAQS.

In December 2006, the EPA announced a revised procedure for evaluation of the NAAQS that consists of four major components: Planning, Integrated Science Assessment, Risk/Exposure Assessment, and Policy Assessment/Rulemaking (http://www.epa.gov/ttn/naaqs/memo_process_for_reviewing_naaqs.pdf). The Planning process requires preparation of an integrated, policy-relevant plan that would follow the completion of the prior NAAQS review. The Integrated Science Assessment effort will provide a concise evaluation, integration, and synthesis of the most policy-relevant science, including key science judgments that would be used in conducting the risk and exposure assessments. The Risk/Exposure Assessment will develop a concise risk/exposure assessment document focused on key results, observations, and uncertainties. Following the Integrated Science Assessment and the Risk/Exposure Assessment, the Agency will develop a Policy Assessment/Rulemaking report that reflects the Agency's view on whether the current standard should be retained or revised. This report replaces the Staff Paper. CASAC review continues throughout the entire review process.

In 1996, EPA staff recommended a separate secondary standard for vegetation. However, CASAC could not agree on the form of a secondary standard. Since the primary standard recommended during that assessment was thought to be sufficiently protective of vegetation, the secondary standard remained the same as the primary. The current U.S. NAAQS for $O_3$ is concentration based, 0.08 ppm (0.084 ppm and below) 8-h average, fourth highest daily maximum concentration averaged over a 3-year period. Similarly, concentration-based air quality standards for $O_3$ exist in Canada and Mexico. The North American air quality standards are based on not-to-exceed $O_3$ concentration values. They do not preferentially weight the higher concentrations, are not cumulative over a growing season, and do not consider stomatal uptake, factors important in plant response. A recent U.S. National Academy of Sciences Report[22] indicates "EPA's current practice for setting secondary standards for most criteria pollutants does not appear to be sufficiently protective of sensitive crops and ecosystems."

The EPA has recognized that the hourly $O_3$ concentrations, 8-h averages, or seasonal averages of the hourly concentrations may not be adequate $O_3$ metrics for protecting vegetation, and that peak $O_3$ concentrations and cumulative exposure are important in plant response[10,23]. The EPA recognizes that the current concentration-based primary ambient air quality standard for $O_3$ in the U.S. may not adequately protect vegetation. On August 25, 2006, CASAC recommended to the EPA Administrator that the maximum 3-month, 12-h (0800–1959 h) W126 exposure index be used as the $O_3$ secondary standard. Although the EPA also understands the usefulness of flux-based metrics for relating ambient $O_3$ to plant response, it believes that there is insufficient scientific information available at this time to apply a flux-based metric in the U.S. Data are lacking for plant species in North America for parameterization of flux models. Musselman et al.[1] have recognized the usefulness of flux-based metrics in plant response to $O_3$, but also concluded that the current lack of data available to use effective flux limits the practicality of using flux-based metrics for $O_3$ standards. However, Musselman et al.[1] recommended that a 24-h cumulative exposure metric be used to relate plants effects to ozone exposure.

Although the current U.S. NAAQS concentration metrics are not biologically based in terms of parameters related to vegetation effects, it has been reported that the 8-h average concentration-based standards do perform well compared to the exposure-based metrics SUM00 and AOT40 for yield of aspen and birch[24]. The authors were careful to note that none of the $O_3$ parameters examined showed a statistically significant relationship to growth and should not be used alone to predict growth[24]. Percy et al.[25] combined the fourth highest 8-h average concentration experienced in their high exposure chambers with other parameters and found a statistically significant result. It is important to note that those study conclusions were derived with data from fumigation exposures with high hourly average concentrations in the experimental chambers and the 8-h average concentrations may be highly correlated with the frequent occurrences of these elevated levels. This implies that at actual locations in the U.S. and Canada that experience infrequent occurrences of elevated hourly average $O_3$ concentrations, their model may not adequately relate the fourth highest 8-h average concentrations with effects.

The SUM00 cumulative exposure index is similar to a long-term average $O_3$ concentration parameter because it sums all $O_3$ concentrations and would not be expected to relate well with vegetation effects. The AOT40 has not performed well for predicting yield effects[12]. Since the AOT40 metric accumulates hourly average concentrations above 40 ppb, it cannot differentiate between the occurrences of high- and mid-level concentrations. Thus, the AOT40 accumulated values experienced at high-elevation monitoring sites are large because of the numerous hourly average concentrations in the mid levels that occur on a continuous 24-h basis. Although one might predict large growth losses associated with the high AOT40 values, they are unlikely to occur because of the absence of the elevated hourly average concentrations. This results in the lack of correlation between the AOT40 values and vegetation effects.

The EPA is now exploring the critical loads and levels approach to assess nitrogen, sulfur, and $O_3$ impact on ecosystems[22], separate from the EPA effort to establish NAAQS. Even though the U.S. has not adopted an exposure-based, flux-based, or effective flux-based standard to determine $O_3$ effects on vegetation, some federal agencies are using exposure-based parameters to evaluate the effects of $O_3$ on natural ecosystems[26,27]. Tools are available online for converting hourly average $O_3$ data to the SUM06 and W126 values (http://webcam.srs.fs.fed.us/calculator/calculator.htm).

Currently, very little critical levels research is underway in North America. Critical loads are beginning to be calculated for ecosystems in the U.S. and Canada, but data are not available for calculating flux-based or effective flux-based critical levels for $O_3$ for North American crops, trees, and natural vegetation. It is expected that progress will continue in critical loads analysis in North America, using the SMB and VSD models for terrestrial ecosystems and the MAGIC model for aquatic ecosystems; but little progress is expected to be made in critical levels analysis.

Future research should focus on (1) the development of data that are needed to parameterize flux models, particularly lacking for North American species; and (2) better quantifying the important aspects of detoxification. The detoxification information must be integrated with flux-based models so that effective flux models can adequately describe the important relationships among concentration, update, and temporally related detoxification. It is important that researchers continue to focus on the importance of nighttime exposure and its relationship to update and detoxification. Current flux-based models, because of temporally related detoxification processes, cannot adequately describe the relationship between effective dose and vegetation effects, even with the application of flux-based thresholds. Thus, until a second generation of effective flux-based models is produced, cumulative exposure indices, coupled with numbers of hourly averages $\geq$ 0.10 ppm, and the Palmer hydrologic index[9,28,29] may offer better predictive capabilities than current exposure-based indices used alone.

## SUMMARY

Ozone parameters that are most closely related to vegetation response are cumulative, preferentially weight the peaks, and occur during time periods when stomata are open, facilitating uptake into plant tissue[10,23]. Air quality standards in North America are concentration based, dependent on the highest hourly or 8-h average $O_3$ concentrations as standards for effects on vegetation, but are not cumulative nor do they consider plant uptake. European assessments of $O_3$ effect on vegetation are exposure based, and more recently for limited numbers of crops, flux based. The European AOT40 parameter is cumulative, weights the peaks to some extent by eliminating the lowest concentrations, and ignores the nighttime concentrations. The AOT40 parameter has limitations that have been documented in the literature. There is progress toward adapting a flux-based assessment in Europe, but there are still inadequate data on effective flux, limiting the usefulness of flux-based metrics in determining vegetation response to $O_3$. Until the effective flux-based models are implemented, the best predictors of plant response to $O_3$ appear to be the cumulative exposure-based indices linked with a quantification of peak concentrations and environmental variables, such as a soil moisture index.
